# Prevalence of vaccination against the Covid19 within mentally ill population and acceptance and hesitancy factors

**DOI:** 10.1192/j.eurpsy.2022.1386

**Published:** 2022-09-01

**Authors:** K. Makni, L. Zouari, R. Feki, N. Smaoui, S. Omri, I. Gassara, M. Maalej, J. Ben Thabet, N. Charfi, M. Maalej

**Affiliations:** 1 University Hospital of HediChaker, Psychiatry C Department, Sfax, Tunisia; 2 Hedi Chaker University Hospital, Psychiatry C, Sfax, Tunisia; 3 Hedi Chaker University Hospital, Psychiatry, Sfax, Tunisia

## Abstract

**Introduction:**

Vaccines are effective interventions that can reduce the high burden of COVID19 globally. However, public vaccine hesitancy is a pressing problem for public health authorities.

**Objectives:**

This study aimed to assess the prevalence of vaccination within mentally ill population and to point out the factors of acceptance and reticence.

**Methods:**

We conducted a cross-sectional, descriptive and analytical study. It was carried out on a clinical population who consult in the psychiatry department in Sfax’s university hospital Hedi Chaker. Patients included in our study were aged between 21 and 69 years and were not in a decompensation phase of their psychiatric illness

**Results:**

Forty five patients were included. The mean age was 45±13 years old. Our population was made up of 3 women (6.7%) and 42 men (93.3%).A rate of 42.2% of the patients was of urban origin, 15.6% lived with a partner, 77.8% were unemployed and 46.7% were schizophrenic. In our study population, five patients had COVID 19 (11.1%), fourteen patients (31.1%) were vaccinated and eight patients (17.8%) asked their psychiatrist to vaccine. The main reasons of vaccination were their belief that vaccination decreases the chance of contracting COVID 19 and its complications (0.00) ,that COVID is lethal (0.002), and the fact that they trust it (0.001). Thirtyone patients (68.9%) refused vaccination, mainly due to reading or hearing negative information about vaccination (0.025). Vaccination wasn’t correlated neither to the fact that it could be a conspiracy nor to the diagnostic.

**Conclusions:**

Our study reveals that one third of mentally ill patients are vaccinated. The reasons of acceptance of vaccination are multiple in opposite of the hesitancy factors

**Disclosure:**

No significant relationships.

